# Parasitic infections and maternal anaemia among expectant mothers in the Dangme East District of Ghana

**DOI:** 10.1186/s13104-016-2327-5

**Published:** 2017-01-03

**Authors:** Samuel Crowther Kofi Tay, Emmanuel Agbeko Nani, Williams Walana

**Affiliations:** 1 Department of Clinical Microbiology, School of Medical Sciences, Kwame Nkrumah University of Science and Technology, Kumasi, Ghana; 2 Department of Clinical Microbiology, School of Medicine and Health Sciences, University for Development Studies, Tamale, Ghana

**Keywords:** Parastic, Maternal, Anaemia, Ghana

## Abstract

**Background:**

Parasitic infections are of public health concern globally, particular among at risk groups such as pregnant women in developing countries. The presence of these parasites during pregnancy potentiate adverse effects to both the mother and the unborn baby. This study sought to establish the prevalence of some parasitic agents among antenatal attendees in the Dangme East District of Ghana. A cross-sectional prospective study was conduct between April and July, 2012. Venous blood specimens were collected from each participant for haemoglobin estimation and malaria microscopy. In addition participants’ early morning mid-stream urine and stool specimens were analyzed microscopically for parasitic agents.

**Results:**

A total of 375 pregnant women were involved in the study, of which anaemia was present in 66.4% (249/375). However, parasitic infections associated anaemia prevalence was 49.6% (186/375). In all, 186 cases of parasitic infections were observed; 171 (44.0%) were single isolated infections while 15 (4.0%) were co-infections. *Plasmodium* species were significantly associated with anaemia (13.3%, χ^2^ = 23.290, *p* < 0.001). Also, the presence of *Schistosoma haematobium* (3.7%, χ^2^ = 7.267, *p* = 0.008), *Schistosoma mansoni* (5.3%, χ^2^ = 5.414, *p* = 0.023) and hookworm (3.7%, χ^2^ = 11.267, *p* = 0.008) were significantly associated with anaemia in pregnancy. Except where co-infections exist (3.7%, χ^2^ = 11.267, *p* = 0.001), the rest of the single infections were insignificantly associated with anaemia. Collectively, intestinal helminthes were predominantly significant with anaemia in pregnancy (*p* = 0.001, χ^2^ = 107.800).

**Conclusion:**

The study revealed relatively high prevalence of parasitic infections among the study population, suggesting that about three-quarters of the anaemic mothers are either single or co-infected with parasitic agents.

## Background

Parasitic infections are of general public health concern globally. The effects of such infections among pregnant women is even more enormous, with adverse outcomes including low pregnancy weight gain and intra-uterine growth retardation (IUGR), which breeds low birth weight (LBW) [1, 2]. Such infections contribute to poor nutritional status, anaemia, and impaired growth, and may culminate to congenital anomalies in new-borns and in some cases mothers may die from them. Parasitic infections have no global boundaries [3]. However, their prevalence are higher in the tropical and sub-tropical regions of the globe where ambient conditions favour their survival and transmission, particularly impoverished areas which is usually coupled with poor personal and environmental hygiene.

Globally, an estimated 3.8 billion persons are infected with geo-helminth, with an annual clinical case and complications associated mortality rate of approximately 720 million and 135,000 respectively. There are 800–1000 million cases of *Ascaris lumbricoides*, 700–900 million cases of hookworm (*Necator americanus* and *Ancylostoma duodenale*), and 500 million cases of *Trichuris trichiura* [4, 5]. Although acute symptoms of these infections are uncommon, numerous studies suggest consistent association between intestinal infections and diminished food intake and weight loss 2010 [6].

An estimated ten million pregnant women in Africa who are infected with schistosomiasis suffer from anaemia and almost seven million pregnant women in sub-Saharan Africa are infected with hookworms and are also at risk of developing anaemia [6, 7]. *Schistosoma haematobium* causes urogenital schistosomiasis in about one third of infected women, and this is considered a potential risk factor for sexually transmitted infection and adverse outcome of pregnancy [8, 9]. More than 90% of schistosomiasis cases are found in sub-Saharan Africa and more than 200,000 deaths are attributed to this infection in Middle East and North Africa [10, 11]. In Sub-Saharan Africa, malaria in pregnancy contributes to 15% of maternal anemia, 14% of low birth weight (LBW) infants, 30% of preventable LBW, 70% of intrauterine growth retardation, 36% of premature deliveries, and 8% of infant mortality [12].

Pregnancy has been reported as a major factor that compromises the immune response of women, and make them susceptible to most parasitic infections [13]. However, the prevalence of preventable parasitic infections such as intestinal helminthiasis, schistosomiasis, and malaria, which potentiate blood lost, and cause higher prevalence of anaemia in pregnancy compared with non-pregnant women, should be a course of concern. Even though there exist efforts aimed at preventing parasitic infections in pregnancy, the challenge still persist, particularly in deprived communities and among impoverished individuals. This study therefore sought to determine the prevalence of these parasitic agents and their association with maternal anaemia among rural pregnant women in the Dangme East District of Ghana.

## Methods

### The study area

The Dangme East District is situated in the eastern part of the Greater Accra Region of Ghana and has Ada-Foah as its capital. It shares common boundaries with North Tongu district at the North, South Tongu district and Dangme West at the East and West respectively. At the south is the Gulf of Guinea, which stretches over 45 km. The district lies within latitudes 5°45 south and 6°00 north and from longitude 0°20 west to 0°35 east. The district forms part of the south-eastern coast plains of Ghana, which is one of the hottest part of the country. The average rainfall is about 750 mm per year. Temperatures are high throughout the year and range between 23 and 33 °C during the hot season. Humidity is very high; about 60% due to its proximity of the sea, the Volta River and other water bodies. Daily evaporation rates ranges from 5.4 to 6.8 mm. The vegetation is generally coastal savannah, characterized by short savannah grass interspersed with shrubs and short trees. Along the stretch of the sea shores are few mangrove trees and coconut groves. The savannah also provides extensive land for grazing livestock. Majority of the populace are engaged in livestock production, fishing, cash crop farming and trading. The district has an estimated population of 130,795 persons, and the population under study consisted of pregnant women visiting the Sege Health Centre, Bonikope Health Centre and Anyamam Health Centre, all within the Dangme East district.

### Sampling and data collection

This was a cross sectional study conducted in the Sege Health Centre, Bonikope Health Centre and Anyamam Health Centre. The study population consisted of pregnant women visiting the health centres between April and July, 2012. A total of 375 pregnant women seeking antenatal care for the first time, who voluntarily accepted to be part of the study were randomly enrolled. Participants’ socio-demographic characteristics including age, gravida and parity were documented. Venous blood, early morning mid-stream urine, and stool samples were also collected for laboratory investigations.

### Haemoglobin estimation

Haemoglobin levels of participants were measured using Sysmex haemoglobin analyzer (Sysmex KX-21 N, Sysmex Corporation KOBE Japan). Following the standardization of the analyzer, blood samples obtained in ethylenediaminetetraacetic acid (EDTA) anticoagulant tubes were run for haemoglobin levels.

### Malaria parasitaemia estimation

Participants’ blood were examined microscopically to estimate malaria parasites level per microliter of blood using Giemsa’s staining technique. Thick and thin smears were prepared on clean, dry microscope glass slides and allowed to dry. The thin smears were fixed in absolute methanol and both smears were stained with 2% Giemsa (BDH Laboratory Supplies, Poole BH15 ITD, England). Malaria parasites were identified and enumerated in accordance with the WHO standard [14]. The results were recorded as ‘present’ for positive slide or ‘absent’ for negative slides.

### Stool examination

The formol-ether concentration technique was employed to examine the stool samples for parasitic helminthes as described by Cheesbrough [15]. Briefly, with the aid of an applicator stick, about 1 g of stool sample was emulsified in 3–4 ml of 10% formalin and the content transferred into 10 ml centrifuge tube. The contents were mixed by shaking for 20 s and then sieved into a beaker. The sieved suspension were poured back into the centrifuge tube and the debris discarded. Equal volume of diethyl-ether was added to the suspension in the tube and was well stoppered, mixed rigorously and opening the stopper intermittently to discharge the gas. The content was centrifuged at 3000*g* for 1 min. The supernatant was decanted in a single movement into a bowl containing disinfectant; allowing the last few drops of residual fluid to flow back onto the sediment and the tubes placed in a rack. The sediment was re-suspended with a disposable Pasteur pipette and a few drops transferred onto a microscope slide, and covered with a cover slip. The specimen was examined microscopically using the low power (×10) objective, in a systematic manner ensuring observation of the entire coverslip area. A higher magnification (×40) objective was used to observe the detailed morphology of ova or larvae found under the light microscope (Olympus CX21FSI).

### Urine examination

The membrane filtration technique was used to concentrate urine specimens for *Schistosoma haematobium* eggs as previously described by Cheesbrough [15]. Each specimen was homogenized and 10 ml aliquot pushed through 8 µm filter membrane fitted in a 25 mm Millipore filter. The process was repeated using 10 ml of physiological saline. The filter holder was disengaged and the filters removed and placed upside down onto a microscope slide. A drop of physiological saline was added to moisten the filtrate. Slides were examined under the microscope for *Schistosoma haematobium* eggs using both ×10 and ×40 objectives. The number of eggs were counted and was reported per 10 ml of urine.

### Data analysis

Data entry and validation was performed in excel, and statistical analysis was done using Statistical Package for Social Sciences (SPSS) version 16.0. Values were considered statistically significant when *p* values were less than 0.05. Chi square was used to determine the association between hemoglobin concentrations and infections, age group and infections, gravidity and infections, and parity and infections as indicators of anaemia. Binary logistic regression was used to determine the odds at which an infection could cause anaemia. Cross tabulations was used to determine the frequencies and percentages between variables.

## Results

### Socio-demographic characteristics and anaemia distribution

A total of 375 pregnant women were included in the study. Majority of the participants were within the age bracket 20–29 years 57.6% (216), followed by those aged 30–39 years 23.2% (87). Teen age mothers were the third common category of pregnant women 16.5% (62) and the least group were mothers aged 40 years and above 2.7% (10). Anaemic pregnancy was present in 66.4% (249/375) of the women. Anaemia was predominantly present among expectant mothers in the age group 20–29 years 37.9% (142), and the least dominant occurred in mothers ≥40 years old 1.6% (6). Classifying the participants into gravida, majority were multigravida 56.8% (213/375), and anaemia 35.5% (133/375) was dominant in same, followed by primagrivida 17.6% (66/375). Grouping participant by parity, the domineering group was multiparous 52.0% (195/375) with predominating anaemia of 32.5% (122/375). Anaemia was however least dominant among nulliparous women (16.3%). Generally, anaemia was significantly associated with age, gravidity and parity (Table 1).

**Table 1:** Socio-demographic characteristics and anaemia distribution among the pregnant women

Characteristics	Total		Anaemia					
			Absent (n = 126; % = 33.6)		Present (n = 249; % = 66.4)		χ^2^	*p * value
	N	%	n	%	n	%		
Age (years)								
15–19	62	16.5	14	3.7	48	12.8	157.600	<0.001
20–29	216	57.6	74	19.7	142	37.9		
30–39	87	23.2	34	9.1	53	14.1		
40–49	10	2.7	4	1.1	6	1.6		
Gravidity								
Primigravid	93	24.8	27	7.2	66	17.6	46.723	<0.001
Secondagravid	69	18.4	19	5.1	50	13.3		
Multigravid	213	56.8	80	21.3	133	35.5		
Parity								
Nulliparous	88	23.5	27	7.2	61	16.3	27.639	<0.001
Primiparous	92	24.5	26	6.9	66	17.6		
Multiparous	195	52.0	73	19.5	122	32.5		

### Association between parasitic infections and maternal anaemia

In all, 186 cases of infections were observed, of which 171 (44.0%) were single isolated infections while 15 (4.0%) were co-infections. The prevalence of the parasites identified were *Plasmodium* species 62 (16.5%), Hookworm 15 (4.0%), *Schistosoma haematobium* 17 (4.5%), *Ascaris lumbricoides* 32 (8.5%), *Trichuris trichiuria* 22 (5.9%), *Schistosoma mansoni* 28 (7.5%), *Strogyloides stercoralis* 7 (1.9%) and *Taenia* specie*s* 3 (0.8%). Comparing parasitic infestation with the presence of anaemia, *Plasmodium* species were significantly associated with maternal anaemia (13.3%, χ^2^ = 23.290, *p* < 0.001). Also, the presence of *Schistosoma haematobium* (3.7%, χ^2^ = 7.267, *p* = 0.008), *Schistosoma mansoni* (5.3%, χ^2^ = 5.414, *p* = 0.023) and hookworm (3.7%, χ^2^ = 11.267, *p* = 0.008) were significantly associated with anaemia in pregnancy. Except where co-infections were observed (3.7%, χ^2^ = 11.267, *p* = 0.001), the rest of the single infections were insignificantly associated with anaemia (Table 2).

**Table 2:** Association between parasitic infections and maternal anaemia among the study population

Infection	Total		Anaemia					
			Absent (n = 126; % = 33.6)		Present (n = 249; % = 66.4)		χ^2^	*p * value
	N = 375	%	n	%	n	%		
*Plasmodium* spp.								
Present	62	16.5	12	3.2	50	13.3	23.290	<0.001
Absent	358	83.5	114	30.4	199	53.1		
*S. haematobium*								
Present	17	4.5	3	0.8	14	3.7	7.118	0.008
Absent	358	95.5	123	32.8	235	62.7		
Hookworm								
Present	15	4.0	1	0.3	14	3.7	11.267	0.001
Absent	360	96.0	125	33.3	235	62.7		
*A. lumbricoides*								
Present	32	9.5	12	3.2	20	5.3	2.000	0.157
Absent	343	91.5	114	30.4	229	61.1		
*T. trichiuria*								
Present	22	5.9	10	2.7	12	3.2	0.182	0.670
Absent	353	94.1	116	30.9	137	63.2		
*S. mansoni*								
Present	28	7.5	8	2.1	20	5.3	5.143	0.023
Absent	347	92.5	118	31.5	229	61.1		
*S. stercoralis*								
Present	7	1.9	2	0.5	5	1.3	1.286	0.257
Absent	368	98.1	124	33.1	244	65.1		
*Taenia* spp.								
Present	3	0.8	1	0.3	2	0.5		
Absent	372	99.2	125	33.3	147	65.9	0.333	0.564
Co-infection								
Present	15	4.0	1	0.3	14	3.7	11.267	0.001
Absent	360	96.0	125	33.3	235	62.7		

*Schistosoma haematobium*, *Ascaris lumbricoides*, *Trichuris trichiuria*, *Schistosoma mansoni*, *Strogyloides stercoralis* and *Taenia* species

### Age, gravidity and parity distributions of parasitic infections

As indicated in Table 3, malaria was significantly associated with age group (*p* < 0.001; χ^2^ = 32.839), gravidity (*p* = 0.001; χ^2^ = 14.742) and parity (*p* = 0.001; χ^2^ = 13.194). The opposite was however seen with respect to *S. haematobium*. Collectively, intestinal helminthes were predominantly significant with age group (*p* = 0.001, χ^2^ = 107.800), gravidity (*p* < 0.001; χ^2^ = 33.347) and parity (*p* < 0.001; χ^2^ = 26.396). However, the individual parasites under intestinal helminthes showed varied associations. Generally, there were no significant association with age group, gravidity and parity, regarding infections with *S. stercoralis*, *Taenia species*, and co-infections. *S. mansoni* and hookworm were significantly associated with age, while *Ascaris lumbricoides* and *T. trichiuria* were significantly associated with age, gravidity and parity.

**Table 3:** Single and co-infections of parasitic agents within age, gravidity and parity

Age groups	15–19 n (%)	20–29 n (%)	30–39 n (%)	40–49 n (%)	χ^2^	*p * value
*Plasmodium* spp.	18 (4.8)	32 (8.5)	11 (2.9)	1 (0.3)	32.839	<0.001
*Schistosoma haematobium*	4 (1.1)	10 (2.7)	3 (0.8)	0 (0.0)	5.059	0.080
Intestinal helminths	*9 (2.4)*	*70 (18.7)*	*16 (4.3)*	*6 (1.6)*	*107.800*	<*0.001*
Hookworm	1 (0.3)	10 (2.7)	3 (0.8)	1 (0.3)	14.600	0.020
*Ascaris lumbricoides*	2 (0.5)	24 (6.4)	6 (1.6)	1 (0.3)	25.750	0.000
*Trichuris trichiuria*	2 (0.5)	12 (3.2)	6 (1.6)	2 (0.5)	12.182	0.007
*Schistosoma mansoni*	3 (0.8)	23 (6.1)	1 (0.3)	1 (0.3)	49.143	0.000
Strongyloides	1 (0.3)	4 (1.1)	0 (0.0)	2 (0.5)	2.000	0.368
*Taenia* spp.	0 (0.0)	3 (0.8)	0 (0.0)	0 (0.0)	0.000	0.166
Co-infection	3 (0.8)	9 (2.4)	3 (0.8)	0 (0.0)	4.800	0.091

Gravidity	Primagravid	Secondagravid	Multigravid		
*Plasmodium* spp.	24 (6.4)	7 (1.9)	31 (8.8)	14.742	0.001
*Schistosoma haematobium*	5 (1.3)	4 (1.1)	8 (2.1)	1.529	0.465
Intestinal helminths	*21 (5.6)*	*19 (5.1)*	*61 (16.3)*	*33.347*	<*0.001*
Hookworm	4 (1.1)	2 (0.5)	9 (2.4)	5.200	0.074
*Ascaris lumbricoides*	4 (1.1)	7 (1.9)	21 (5.6)	15.438	0.000
*Trichuris trichiuria*	4 (1.1)	2 (0.5)	16 (4.3)	15.636	0.000
*Schistosoma mansoni*	7 (1.9)	7 (1.9)	14 (3.7)	3.500	0.174
Strongyloides	1 (0.3)	3 (0.8)	3 (0.8)	1.143	0.565
*Taenia* spp.	1 (0.3)	0 (0.0)	2 (0.5)	0.333	0.564
Co-infection	4 (1.1)	4 (1.1)	7 (1.9)	1.200	0.549

Parity	Nulliparous	Primiparous	Multiparous		
*Plasmodium* spp.	23 (6.1)	8 (2.1)	31 (8.3)	13.194	0.001
*Schistosoma haematobium*	4 (1.1)	7 (1.9)	6 (1.6)	1.529	0.662
Intestinal helminths	*21 (5.6)*	*22 (5.9)*	*58 (15.5)*	*26.396*	<*0.001*
*Hookworm*	4 (1.1)	2 (0.5)	9 (2.4)	5.200	0.074
*Ascaris lumbricoides*	4 (1.1)	8 (2.1)	20 (5.3)	13.000	0.020
*Trichuris trichiuria*	4 (1.1)	3 (0.8)	15 (4.0)	12.091	0.020
*Schistosoma mansoni*	7 (1.9)	7 (1.9)	14 (3.7)	3.500	0.174
*Strongyloides*	1 (0.3)	3 (0.8)	3 (0.8)	1.143	0.565
*Taenia* spp.	1 (0.3)	1 (0.3)	1 (0.3)	0.000	1.000
Co-infection	4 (1.1)	4 (1.1)	7 (1.9)	0.549	0.549

## Discussion

Anaemia in pregnancy is of serious adverse health effect to both the mother and the unborn baby, and its causes are of diverse origin, including both infectious and non-infectious agents. The current study focused on establishing the association between maternal anaemia and parasitic infections among rural expectant mothers in the Dangme East District of Ghana.

The study established pregnancy associated anaemia prevalence of 66.4% (Table 1). However, parasitic infections associated anaemia prevalence was 49.6% (186/375), revealing that about three-quarters (74.7%; 186/249) of the anaemic mothers are either single or co-infected with parasitic agents (Table 2). The current prevalence of anaemia was lower than that reported in Coastal Kenya where 71% of the pregnant women where anaemic [16], but higher when compared to the 53.5% prevalence observed in Southwest Ethiopia [17]. The prevalence noted in this study was comparable to the 62.6% observed in a similar study conducted in Kumasi in the Ashanti region of Ghana, and 61.1% reported in Nigeria [18, 19].

The most predominant parasites associated with the anaemic mothers were *Plasmodium* species (13.3%). However, the overall prevalence of malaria parasite in the study population was 16.5% (Table 2). Malaria in pregnancy has been reported as a major cause of maternal anaemia, particularly in malaria endemic region, hence the observation that most malaria parasite positive mother were anaemic. The malaria prevalence observed was much lower than studies by Yatich et al. and Glover-Amengor et al. [12, 20] which reported a 36.3 and 35.1% respectively among pregnant women in the Ashanti region in Ghana. Other studies in Cameroon [21] and Nigeria [22] have respectively reported 21.9 and 38.8% malaria infection among pregnant women. The relatively low prevalence reported in the present study could be attributed to the fact that the study site falls within the low incidence zone of malaria in Ghana. However, the duration for the current study was in the first raining season in the study area, and relatively high prevalence was anticipated, but the converse was seen. Possibly, the massive educational campaign by the Ghana malaria control program has led to some acquisition of knowledge and subsequently the general public taking practical steps to prevent mosquito bite, particularly among the at risk population.

*Schistosoma haematobium* and hookworm recorded a common prevalence of 3.7% while *S. mansoni* archived a prevalence of 5.2%. The presence of these parasites were strongly associated with maternal anaemia. The overall prevalence of 4.5% for *S haematobium* infection is in consonance with a similar study conducted in Bawku in northern Ghana by Siegrist [23], which reported the same prevalence in a community-based study among pregnant women and outcome of their birth. The association of *S. haematobium* and anaemia agrees with studies by Friedman et al. and Allen [8, 24]. In contrast to this study, McClure [16], reported no association between *Schistosoma haematobium* infection and anaemia among pregnant Kenyan women.

*Schistosoma mansoni* prevalence was 7.5% and the second highest infection in this study. This prevalence fell below a similar study conducted in Northern Ghana which reported 12.3% and another which recorded as high as 47.6% prevalence among women of reproductive age in Uganda [25, 26]. However, prevalence of 7.5% in this study is higher than a study in Nigeria which reported 3.4% and in Benin which reported 0.2%; although the sample size in these two studies were 2104 and 1005 respectively [22, 27]. *Schistosoma mansoni* infection was statistical significance with anaemia and age, but not gravidity and parity. This trend of infection has been previously observed by Muhangi et al. in Uganda [28].

Although hookworm infections are predominant in developing countries, the overall prevalence obtained from this study was 4.0% (Table 2). This finding is similar to 3.9% prevalence obtained among pregnant women in Kenya [29]. The prevalence in the present study was lower than 7.9 and 9.0% obtained respectively by Yatich et al. and Ouedrago in Ghana and Benin, but higher than 0.3% by Walana et al. in Ghana [12, 27, 30]. Even though the general hookworm prevalence was relatively low, its infection was significantly associated with anaemia and age but not gravidity and parity (Tables 2, 3). The prevalence of hookworm among the various age groups were in consonance with a previous study which showed higher prevalence in pregnant women of ≤29 years as compared to their older counterparts [29]. Infection of hookworm classified under gravidity in this study revealed a decline from primigravidae of 1.1 to 0.5% for secondagravidae and a rise to 2.4% for multigravidae, similar to the trend observed by Boel et al. among pregnant women on the Thai- Burmese border [31]. Anaemia caused by hookworm infection was 3.7%. Studies conducted by Melku et al. and McClare et al. in Ethiopia and Kenya respectively also reported similar findings [16, 32]. Poor sanitary disposal of human faeces and indiscriminate defecation are the principal factors in the aetiology of hookworm infections. The low prevalence recorded in the current study could suggest improved sanitation and personal hygience in the study area.

In this study, *Ascaris lumbricoides in* anaemia was 5.3%. This finding was consistent with report by Larocque et al. in Peru [33]. In relation to the age groups, *Ascaris lumbricoides* infection was highest among women in the age bracket 20–29 years; 6.4%, followed by 30–39 years; 1.6%, 15–19 years; 0.3% and 40–49 years. This trend is in consonance with work done in Nigeria by Obiezue et al. [34]. A study conducted among pregnant Kenyan women by Van et al. [35] revealed *Ascaris lumbricodes* prevalence increased with gravidity as observed in the current study; 1.1, 1.9 and 5.6% for primigravidae, secondagravidae and multigravidae respectively. The observation in this study confirms previous findings that pregnancy is associated with increased *Ascaris lumbricoides* and *Trichuris trichiura* infections compared to non-pregnant women [36].

Prevalence of *Strongyloides stercoralis* among the studied pregnant women was 1.9%. This rate is similar to a study in Tanzania among similar population which reported 1.6% and in Ghana which reported 2.3% [25, 37]. The prevalence of *Strongyloides stercoralis* recorded fell below other studies who reported 12.3% in Uganda and 17.9% in Ghana [28, 38]. The low prevalence reported in this present study is consistent with conclusion drawn by Puthiyakunnon et al. who indicated low parasitic level of *Strongyloides stercoralis* [39]. The trend of prevalence among the age groups was similar to that reported by Yatich et al. in Ghana with the infection rate peaking in age group 20–29 years [12]. As observed in similar studies there was no significant association between anaemia and *Strogyloides stercoralis* [33, 40, 41].

In the present study, prevalence of *Taenia* species was 0.8%, which falls within that reported by a study carried out in three rural communities in Cameroon which reported prevalence between 0.4 and 3.0% [42]. Likewise, the study agrees with that of Garcia et al. in Peru who reported 0–1.9% in a community based study [43]. This prevalence is however lower than that by Garcia-Noval et al. which reported a 2.8% in Guatemala [44]. All the *Taenia* spp. infections were observed among participants in the age group 20–29 years. There were however no statistical significance within the age groups and infection with *Taenia* spp. (Table 2). Similarly, *Taenia* spp. infection was not statistical significance within the gravidity, parity and anaemia.

Prevalence of co-infection, thus being infected with more than one parasite was 4.0%. Ivan et al. reported 6.6% co-infection while investigating prevalence of helminth and malaria infections in pregnant HIV-positive Rwandan women receiving anti-retroviral therapy (ART) [45]. The current rate was however thrice lower than that recorded by Yatich et al. who reported 16.6% [12]. Co-infections were only significantly associated with anaemia. Prevalence of co-infection related to gravidity and parity followed the trend 1.1, 1.1 and 1.9% for primigravidae, secondagravidae and multigravidae women respectively. Similar trend was observed for nulliparous, primiparous and multiparous women. This is in contrast with observation made by Ivan et al. (2013) who reported multigravidae women had a lower risk when compared to primigravidae [45]. Mechanisms by which helminthes and malaria affect haemoglobin levels are distinct but their combined presence enhance the risk of anaemia in pregnant women.

## Conclusion

The present study revealed malaria prevalence of 16.5%, urinary schistosomiasis prevalence of (4.5%), stool helminth infection (29.0%) and parasitc co-infection of (4.0%) among the study population. The predominant stool helminth was Ascaris *lumbricoides* (8.5%), followed by *Schistosoma mansoni* (7.5%), *Trichuris trichiura* (5.9%), hookworm (4.0%), *Strongyloides stercoralis* (1.5%) and *Taenia* species (0.8%). Anaemia in pregnancy was significantly associated with age, gravidity and parity. Infection with *Plasmodium* species, *Schistosoma haematobium*, *Schistosoma manoni*, and hookworm were significantly associated with anaemia in pregnancy. Co-infection was also significantly associated with anaemia in pregnancy. It was also revealed that about three-quarters of the anaemic mothers are either single or co-infected with parasitic agents. The study recommends up-scaling public health education to control parasitic infections in the study area. Also, as part of mothers planning to get pregnant, they should be screening for parasitic infections so that infected women can be treated prior to conception. The present study did not take data on socioeconomic, behavioral or environmental factors for analysis. The possible risk factors of parasitic infections in the study area were therefore not covered.

## Abbreviations

IUGR: intra-uterine growth retardation
LBW: low birth weight
EDTA: ethylenediaminetetraacetic acid
WHO: World Health Organisation
SPSS: statistical package for social sciences
CHRPE: Committee on Human Research Publication and Ethics
